# Characteristics of gut microbiota and metabolites in extrahepatic cholangiocarcinoma and their prognostic value for resectable lesions

**DOI:** 10.3389/fcimb.2025.1523863

**Published:** 2025-02-14

**Authors:** Cheng Ye, Bo Zhang, Yanyan Lin, Fangfang Han, Huaqing Shi, Chunlu Dong, Wence Zhou

**Affiliations:** ^1^ The First Clinical Medical College, Lanzhou University, Lanzhou, China; ^2^ Department of General Surgery, The First Hospital of Lanzhou University, Lanzhou, China; ^3^ Department of General Surgery, The Second Hospital of Lanzhou University, Lanzhou, China

**Keywords:** extrahepatic cholangiocarcinoma, gut microbiota, metabolites, postoperative prognosis, predictive value

## Abstract

This study aimed to investigate the relationship between gut microbiota composition, fecal metabolites, and postoperative prognosis in patients with extrahepatic cholangiocarcinoma (eCCA). A total of 53 patients with resectable eCCA and 21 healthy volunteers as a control group were included. 16S rRNA gene sequencing and metabolomic analyses revealed significant differences in the gut microbial community structure and altered fecal metabolites profiles between eCCA patients and healthy controls. Univariate and multivariate Cox regression analyses indicated that factors such as preoperative total bilirubin, indirect bilirubin, and specific metabolites were closely associated with overall survival in patients with eCCA post-surgery. The constructed nomogram model further demonstrated the predictive value of these factors, achieving a C-index of 0.718, with calibration curves confirming its strong predictive performance. In conclusion, gut microbiota composition and fecal metabolites play a crucial role in the surgical prognosis of eCCA patients, providing new insights for clinical prognostic assessment.

## 1 Introduction

Cholangiocarcinoma (CCA) is a malignant tumor originating from the epithelial cells of the bile ducts, usually manifesting as adenocarcinoma. Although its global incidence is relatively low, it has shown a rising trend in certain regions and populations (Patel, 2001; Banales et al., 2016; Saha et al., 2016). According to a study in the United States, from 1973 to 2012, the incidence of intrahepatic cholangiocarcinoma (iCCA) continuously increased, while the incidence of extrahepatic cholangiocarcinoma (eCCA) showed a steady growth (Saha et al., 2016). The clinical symptoms of CCA usually depend on the anatomical location of the tumor. Early stages often present without significant symptoms, while late-stage patients frequently miss the optimal time for surgery due to a long latency period and subtle early symptoms, leading to a 5-year survival rate of less than 20% (Valle et al., 2021; Elvevi et al., 2022). Currently, while early diagnostic markers can help identify CCA at an earlier stage, there is an urgent need for prognostic markers that can predict surgical outcomes and guide postoperative treatment plans, ultimately facilitating personalized treatment approaches for patients with CCA.

In recent years, the role of gut microbiota and their metabolites in the field of oncology has gained increasing attention. Gut microbiota, as a symbiotic microbial community with the host, plays a crucial role in maintaining health, regulating immune function, and balancing metabolism (Ma et al., 2019; Martin et al., 2019; Zheng et al., 2020). Research has shown that dysbiosis of the gut microbiota is closely associated with the occurrence and progression of various gastrointestinal tumors. For example, the presence of Fusobacterium nucleatum DNA in colorectal cancer patients is associated with shorter survival, and it may serve as a prognostic biomarker (Mima et al., 2015, 2016). Additionally, molecules derived from gut bacteria, such as secondary bile acids, lipopolysaccharides (LPS), and lipoteichoic acid (LTA), have been shown to regulate liver antitumor immunity (Dapito et al., 2012; Ma et al., 2018; Singh et al., 2018).

Gut microbiota and their metabolites may play a significant role in the development, progression, and prognosis of CCA. A study by Jia et al. found that in patients with iCCA, an increase in the abundance of certain microbial populations was associated with elevated levels of specific metabolites in plasma, which could potentially serve as biomarkers for differentiating between various types of liver tumors (Jia et al., 2020). Additionally, gut-derived bacteria and LPS may influence the development and progression of CCA by regulating the liver’s immune environment (Zhang et al., 2021a). Therefore, metabolites derived from gut microbiota may impact the prognosis of CCA patients by modulating the host immune system and tumor microenvironment.

This study aims to explore the characteristics of gut microbiota and their metabolites in eCCA patients and to develop a model for predicting the surgical prognosis of eCCA, assessing its impact on patient outcomes. Prognostic biomarkers derived from gut microbiota or fecal metabolites can help identify patients at higher risk of poor outcomes, allowing clinicians to personalize treatment plans, such as optimizing adjuvant therapies and follow-up schedules. This approach could improve survival rates and enhance the quality of life for eCCA patients. Through this research, we hope to provide new prognostic biomarkers and predictive models for the treatment and management of eCCA patients.

## 2 Materials and methods

### 2.1 Participant recruitment

This study included 53 patients diagnosed with eCCA at The First Hospital of Lanzhou University between July 2018 and June 2023. All patients underwent a Multidisciplinary Team (MDT) consultation and were scheduled to receive radical surgical treatment. Additionally, 21 healthy volunteers were recruited as a control group. The diagnosis of eCCA was based on the National Comprehensive Cancer Network (NCCN) guidelines (Benson et al., 2023) and confirmed through histopathology. Exclusion criteria were: (1) age ≤ 18 years; (2) antibiotic or probiotic treatment within the past 8 weeks; (3) prior radiotherapy, chemotherapy, or immunotherapy before surgery; (4) history of inflammatory bowel disease, gastrointestinal obstruction, primary sclerosing cholangitis, or previous digestive tract bypass surgery.

Baseline clinical data were collected from all participants, including gender, age, body mass index, history of hypertension, history of diabetes, complete blood count, biochemical indicators, tumor markers, nerve invasion, microvascular invasion, and tumor differentiation. Follow-up continued until June 1, 2024, with survival information obtained through medical records and telephone interviews to record overall survival (OS) time. This study strictly adhered to the revised Declaration of Helsinki (2008). All enrolled patients and healthy control provided written informed consent. The study protocol was approved by the Ethics Committee of The First Hospital of Lanzhou University (Ref NO. LDYYLL2024-601).

### 2.2 Sample collection, DNA extraction, and 16S rRNA sequencing

Fecal samples from all participants were collected in the early morning. For each sample, 10 grams from the central portion of the stool was collected and divided into three sterile cryogenic tubes within 2 hours. The samples were immediately placed in liquid nitrogen and transferred to a -80°C freezer within 2 hours for storage until DNA extraction.

Genomic DNA from fecal samples was extracted using the MagPure Soil DNA KF Kit following the manufacturer’s protocol. The concentration and purity of the extracted DNA were measured using a NanoDrop 2000 spectrophotometer (Thermo Fisher Scientific, USA) and agarose gel electrophoresis, and the DNA was stored at -20°C for future use. Using the extracted DNA as a template, bacterial 16S rRNA genes were amplified via PCR with barcoded-specific primers and Takara Ex Taq high-fidelity DNA polymerase. The V3-V4 hypervariable regions of the 16S rRNA gene were amplified using the universal primers 343F (5’-TACGGRAGGCAGCAG-3’) and 798R (5’-AGGGTATCTAATCCT-3’) (Nossa et al., 2010) to analyze bacterial diversity. After PCR amplification, the products were checked by agarose gel electrophoresis and purified using AMPure XP magnetic beads. The purified product was used as a template for a second round of PCR amplification. Following further purification with magnetic beads, the final product was quantified using a Qubit fluorometer and adjusted to the appropriate concentration for sequencing. Sequencing was performed on the Illumina NovaSeq 6000 platform, generating 250 bp paired-end reads. All sequencing work was conducted by OE Biotech Co., Ltd. (Shanghai, China).

### 2.3 GC-MS metabolomics analysis

A 30 mg fecal sample was placed in a 1.5 mL centrifuge tube, and two steel beads along with 300 μL of a methanol-water solution (4:1, containing L-2-chlorophenylalanine at 4 μg/mL) were added. After pre-cooling the sample at -40°C for 2 minutes, it was ground at 60 Hz for 2 minutes. Following grinding, 75 μL of chloroform was added, and the sample was subjected to ultrasound extraction in an ice-water bath for 30 minutes. After extraction, the sample was allowed to stand at -40°C for 30 minutes, then centrifuged at 12000 rpm for 10 minutes at 4°C, and 150 μL of the supernatant was transferred to a glass vial. The supernatant was evaporated to dryness, and then 80 μL of a methoxyamine hydrochloride-pyridine solution (15 mg/mL) was added for a 60-minute reaction at 37°C for oximation. Subsequently, 50 μL of BSTFA derivatization reagent and 20 μL of n-hexane were added. The sample was reacted at 70°C for 60 minutes and then left at room temperature for 30 minutes. Finally, metabolomics analysis was performed using the GC-MS platform to obtain the metabolic profile data.

### 2.4 Bioinformatics and statistical analysis

For the 16S rRNA sequencing data analysis, Cutadapt software was used to trim the primer sequences from the raw data. Next, QIIME2 (2020.11) (Bolyen et al., 2019) and the DADA2 (Callahan et al., 2016) algorithm were applied for denoising and chimera removal, followed by deduplication to generate representative sequences and an amplicon sequence variant (ASV) abundance table. Representative ASV sequences were aligned and annotated using the Silva database (version 138) with the default parameters of the q2-feature-classifier plugin. Alpha diversity metrics, including Shannon index, Simpson index, Chao1 index, and Observed_species index, were analyzed with QIIME2, and statistical differences were evaluated using Kruskal-Wallis and Wilcoxon tests. For beta diversity, weighted and unweighted Unifrac principal coordinates analysis (PCoA) was performed. Additionally, Adonis and Anosim tests based on weighted Unifrac distances were used to determine whether significant differences existed between groups. T-tests were employed to analyze differences at the species level. The Linear Discriminant Analysis (LDA) Effect Size (LEfSe) method was used to identify taxa responsible for differences between the microbial communities of the two groups.

The GC-MS raw data in.D format were converted to.abf format using the Analysis Base File Converter software and then processed in MS-DIAL software. The software performed peak detection, identification, deconvolution, alignment, and missing value interpolation, with metabolite characterization based on the LUG database. A three-dimensional data matrix was generated, including metabolite names, retention times, retention indices, mass-to-charge ratios, and signal intensities. Normalization was conducted using internal standards with relative standard deviations (RSD) greater than 0.1 to minimize technical variability. After normalization, redundant peaks were removed, and peak merging was performed to finalize the data matrix for analysis. The matrix was then imported into R software for principal component analysis (PCA) to assess the overall sample distribution and ensure the stability of the analytical process. Orthogonal partial least squares-discriminant analysis (OPLS-DA) and partial least squares-discriminant analysis (PLS-DA) were employed to differentiate metabolic profiles between groups. The variable importance in projection (VIP) scores derived from the OPLS-DA model were used to rank the contribution of each variable to group separation. A two-tailed Student’s T-test was conducted to confirm the significance of differences in metabolite levels between groups. Differential metabolites were identified based on a VIP score >1.0 and p-value <0.05. P<0.05 was considered statistically significant.

## 3 Results

### 3.1 Clinical characteristics of participants

All participants were residents of Northwest China, following a mixed diet. No significant differences were observed between the two groups regarding gender, body mass index (BMI), history of hypertension, diabetes, HBsAg positivity, or cholecystectomy history. However, the eCCA group was significantly older than the healthy control group. The study included 53 patients with eCCA, with 48 (90.6%) diagnosed at TNM stage I-II. Among them, 12 cases were hilar cholangiocarcinoma, and 41 were distal cholangiocarcinoma (dCCA). The median levels of CA19-9, carcinoembryonic antigen (CEA), and alpha-fetoprotein (AFP) were 147.5 mg/L, 2.3 mg/L, and 2.2 mg/L, respectively. A detailed summary of participants’ general characteristics and clinical parameters is provided in **Table 1**.

**Table 1:** Basic information and clinical characteristics of the participants.

Characteristics	eCCA (n=53)	Healthy control (n=21)	*P* value
Male, n (%)	35 (66%)	12 (57.1%)	0.474
Age (year)	60.00 (33-77)	49 (19-70)	0.001
BMI, kg/m^2^	22.857 (17.631-28.578)	23.588 (17.916-27.681)	0.973
Tbil, μmol/L	213.8 (8.1-720.1)	–	–
Ibil, μmol/L	79 (6.3-257.7)		
ALP, U/L	418.6 (8.6-2856.5)	–	–
GGT, U/L	750.3 (83.6-2209.5)	–	–
Total bile acid	120.2 (1.1-569.7)	–	–
AFP, μg/L	2.2 (1.00-9.10)	–	–
CA199, μg/L	147.5 (0.90-1000.0)	–	–
CEA, ng/mL	2.3 (0.70-89.00)	–	–
Hypertension, n (%)	13 (24.5%)	2 (9.5%)	0.148
Diabetes, n (%)	4 (7.5%)	1 (4.8%)	0.667
cholecystectomized, n (%)	3 (5.7%)	3 (14.3%)	0.220
HBV-infected, n (%)	3 (5.7%)	1 (4.8%)	0.878
dCCA, n (%)	41 (77.4%)	–	–
TNM stage (I-II), n (%)	48 (90.6%)	–	–
Dietary habit	Mixed diet	Mixed diet	–

BMI, Body Mass Index; Tbil, Total bilirubin; Ibil, Indirect bilirubin; ALP, alkaline phosphatase; GGT, Gamma-glutamyl transpeptidase; AFP, Alpha-fetoprotein; CEA, Carcinoma Embryonic Antigen; dCCA, distal cholangiocarcinoma.

### 3.2 Altered diversity of gut microbiota in patients with eCCA

**Figure 1:**
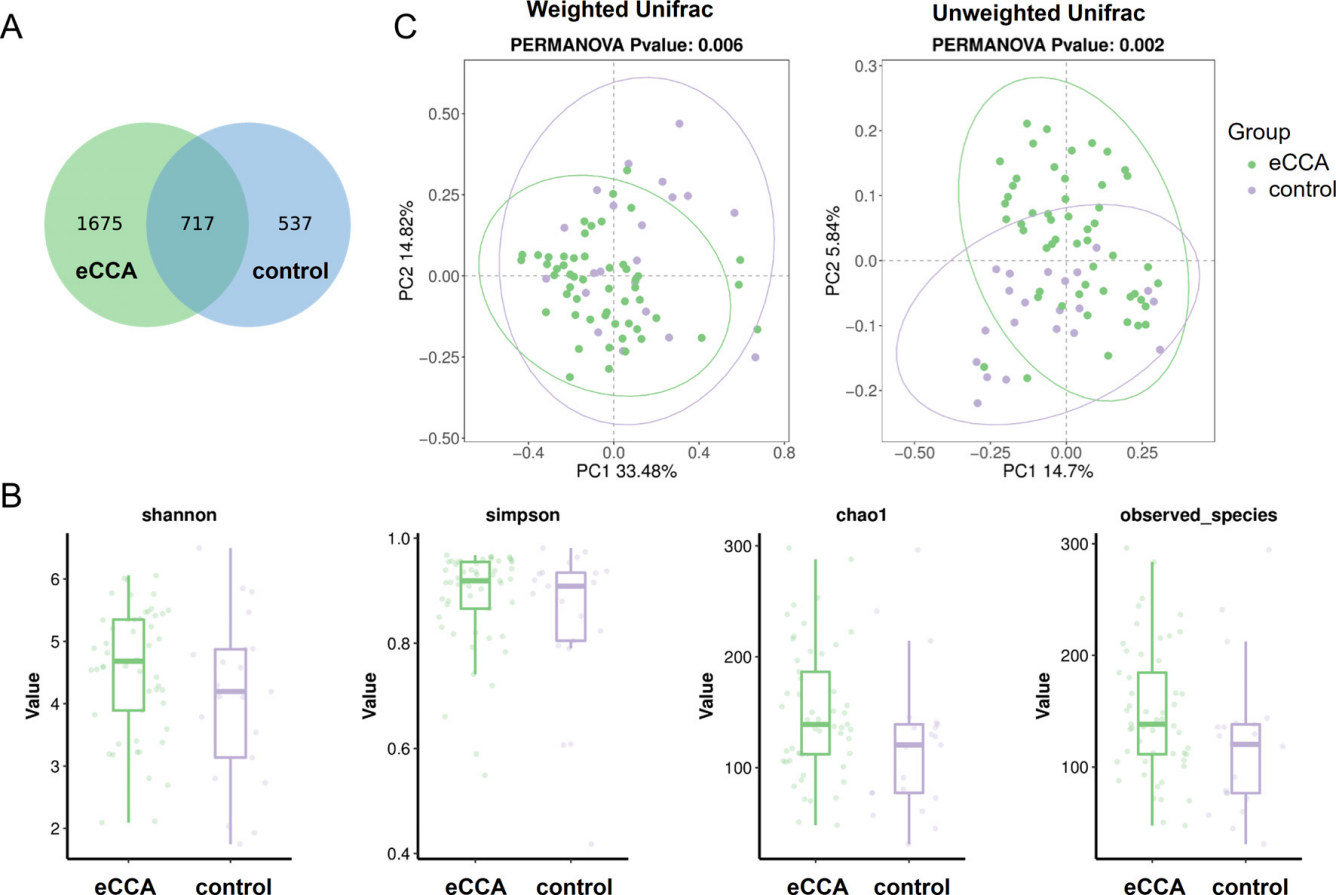
Differences in Gut Microbiota Between Patients with eCCA and the Control Group. **(A)** Venn diagram showing the overlap of ASVs between the two groups. **(B)** Boxplots illustrating the differences in gut microbiota diversity between the two groups based on Shannon, Simpson, Chao1, and Observed_species indices. **(C)** Principal Coordinates Analysis (PCoA) plots based on unweighted and weighted UniFrac distances, demonstrating the distributional differences in gut microbiota between the two groups.

This study generated a total of 4,167,375 high-quality 16S amplicon sequencing reads, with an average of 56,316 sequences per fecal sample. The Venn diagram (**Figure 1A**) identified 2,929 ASVs across all samples, with 2,392 ASVs in the eCCA group and 1,254 ASVs in the healthy control group. Of these, 717 ASVs were shared between the two groups (**Supplementary Table S1**). We further analyzed the alpha diversity of the gut microbiota, comparing the eCCA group with the healthy control group. The results showed no significant differences between the two groups in terms of the Shannon, Simpson, Chao1, and Observed_species indices (**Figure 1B**). However, PCoA based on weighted Unifrac distances revealed that the gut microbiota in eCCA patients was more clustered and significantly distinct from that of the healthy control group (P = 0.006). Similar trends were observed in the PCoA analysis using unweighted Unifrac distances (P = 0.002) (**Figure 1C**). Additionally, Adonis analysis (P = 0.006) and Anosim analysis (P = 0.019) using weighted Unifrac distances further confirmed significant differences between the two groups. In summary, the gut microbiota diversity in patients with eCCA exhibited notable alterations compared to that of the healthy control group.

### 3.3 Differences in gut microbiota composition at the phylum and genus levels in eCCA patients

**Figure 2:**
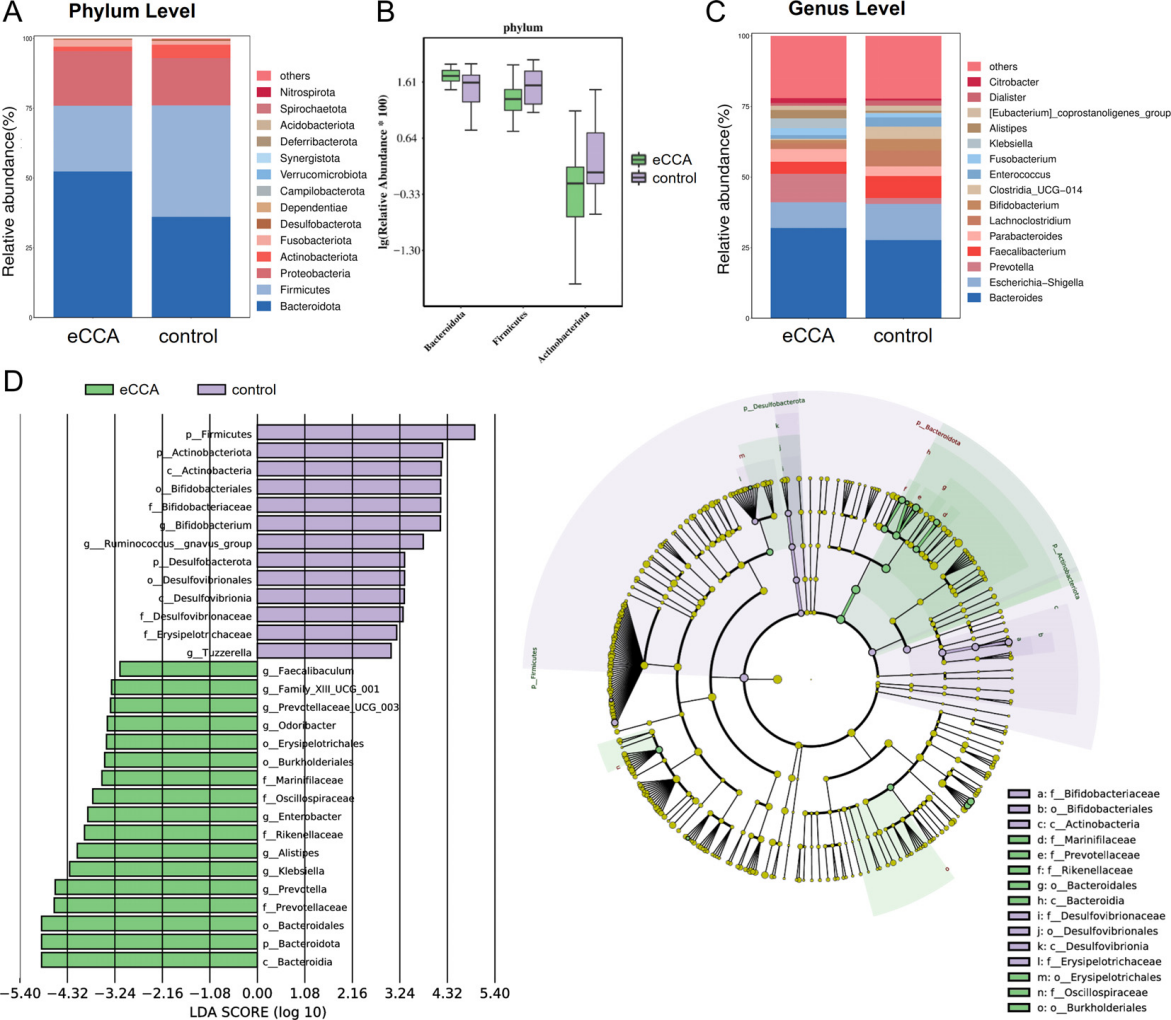
Differences in gut microbiota composition between patients with extrahepatic cholangiocarcinoma and the control group. **(A**, **C)** Bar charts showing the distribution of gut microbiota composition at the phylum level **(A)** and genus level **(C)** in both groups. **(B)** Box plot illustrating the relative abundance of different gut microbiota at the phylum level. **(D)** Linear Discriminant Analysis (LDA) Effect Size (LEfSe) analysis presented as a histogram and cladogram depicting significantly enriched taxa between the two groups.

At the phylum level, 14 microbial phyla were identified. Among them, *Bacteroidetes*, *Firmicutes*, *Proteobacteria*, *Fusobacteria*, and *Actinobacteria* were the dominant phyla in both groups, with their relative abundance exceeding 1%. In patients with eCCA, *Bacteroidetes* was the most abundant phylum (average abundance: 52.36%), followed by *Firmicutes* (23.55%) and *Proteobacteria* (19.55%), with these three phyla accounting for over 95% of the total microbial community (**Figure 2A**). Notably, the abundance of *Bacteroidetes* in the eCCA group was significantly higher than in the healthy control group (P = 0.003), while *Firmicutes* (P = 0.002) and *Actinobacteria* (P = 0.017) were significantly lower in the eCCA group, with statistical significance (**Figure 2B**). At the genus level, 279 microbial genera were identified, with distinct differences in the microbial distribution between the eCCA and healthy control groups (**Figure 2C**). LEfSe analysis revealed 11 genera with significant differences in abundance between the two groups (**Figure 2D**). Specifically, *Klebsiella*, *Alistipes*, *Enterobacter*, *Prevotella*, *Prevotellaceae_UCG_003*, *Faecalibaculum*, *Odoribacter*, and *Family_XIII_UCG_001* were enriched in the eCCA group, while *Bifidobacterium*, *Ruminococcus gnavus group*, and *Tuzzerella* were more abundant in the healthy control group. In conclusion, the gut microbiota composition of eCCA patients shows significant differences from that of the healthy control group at both the phylum and genus levels.

### 3.4 Metabolite alterations in eCCA group

**Figure 3:**
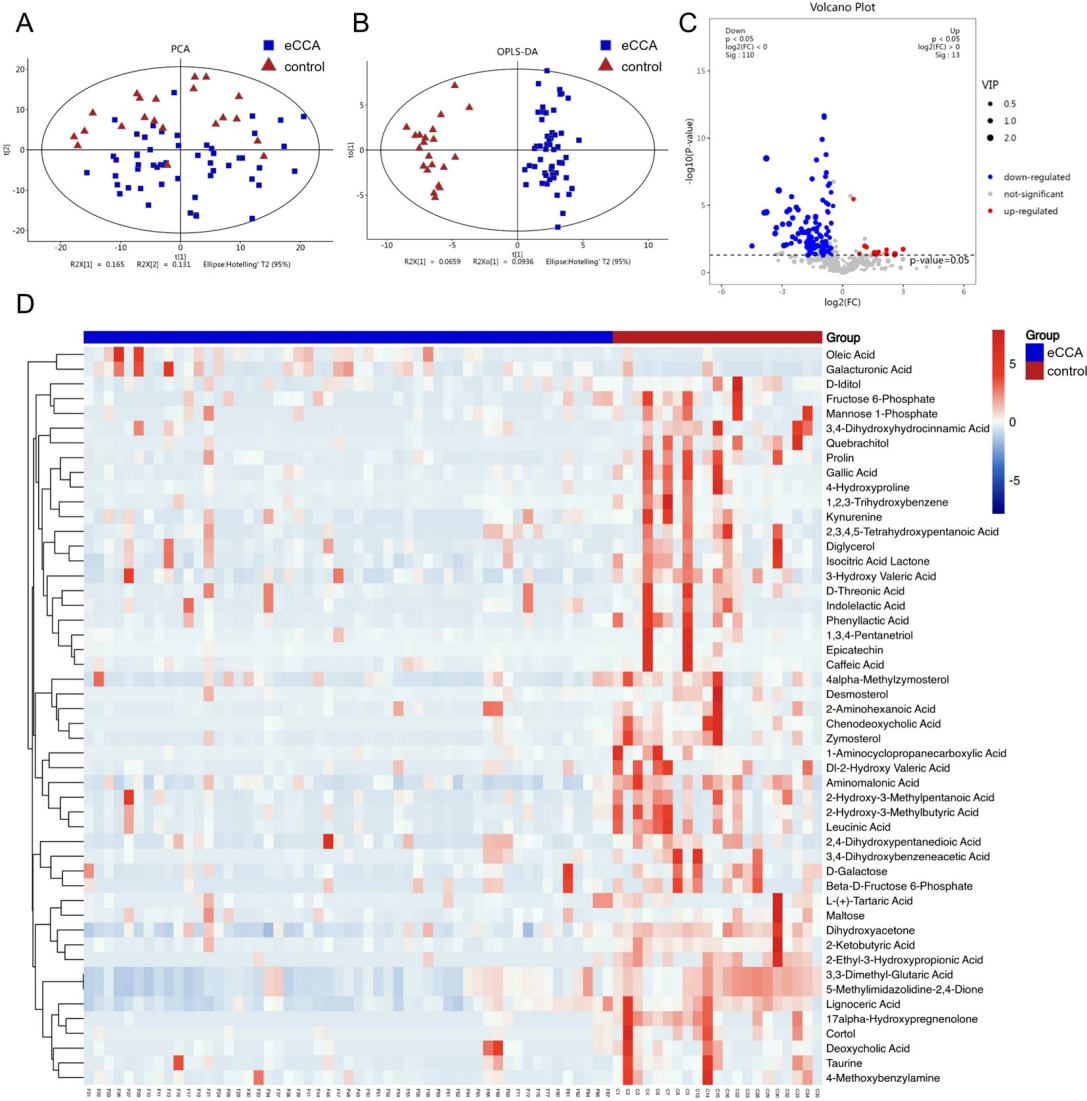
Differences in gut microbiota metabolites between eCCA and the control group. Principal Component Analysis (PCA) **(A)** and Orthogonal Partial Least Squares Discriminant Analysis (OPLS-DA) **(B)** showing the differences in fecal metabolites between the two groups. **(C)** Volcano plot of differential fecal metabolites (red dots indicate significantly upregulated metabolites in the eCCA group (p<0.05, VIP>1, and F/C>1), blue dots indicate significantly downregulated metabolites (p<0.05, VIP>1, and F/C<1), and gray dots indicate non-significant metabolites). **(D)** Cluster heatmap of differential fecal metabolites (top 50 differential metabolites).

The metabolite data matrix of all participants is shown in **Supplementary Table S2**. We conducted principal component analysis (PCA) on the metabolites from the eCCA group and the healthy control group. The results showed that the samples in the eCCA group formed distinct clusters, differing from the control group, with some samples exhibiting dispersion. This indicates that there are differences in the gut microbial metabolites between the two groups (**Figure 3A**). To further emphasize the differences, we performed OPLS-DA. The results revealed a significant separation between the two groups, indicating good classification performance and significant metabolic differences (**Figure 3B**). To prevent overfitting of the OPLS-DA model, we employed seven-fold cross-validation and 200 response permutation tests (RPT) to evaluate the model’s quality. As shown in **Supplementary File S1**, the R²Y and Q²Y values were 0.771 and 0.624, respectively, indicating that the model possesses a certain degree of interpretability and predictive ability. A total of 521 metabolites were detected, and volcano plots were used to visualize the P-values, VIP scores, and fold change (F/C) values, helping to identify differential metabolites. The analysis identified 123 significantly altered metabolites, with 13 upregulated and 110 downregulated in the eCCA group (**Figure 3C**). Additionally, a heatmap (**Figure 3D**) displayed the top 50 metabolites with the most significant differences, most of which showed a downregulated trend in the eCCA group.

### 3.5 Cox regression analysis of factors affecting postoperative overall survival in eCCA

**Figure 4:**
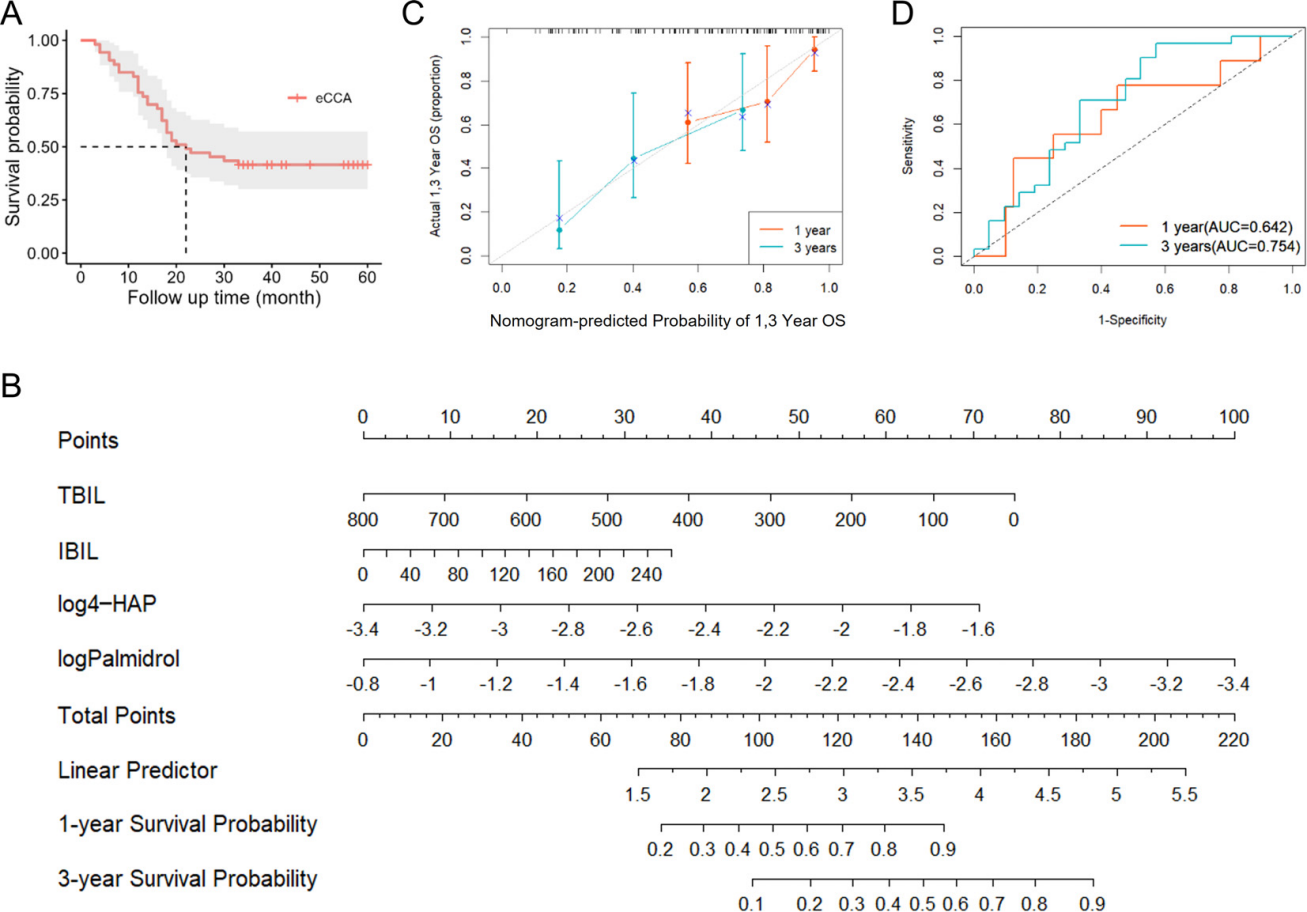
Prognostic analysis of patients with eCCA. **(A)** Survival curve of patients with eCCA. **(B)** Nomogram model constructed based on prognostic factors in the study cohort of eCCA patients. **(C)** Calibration curve of the nomogram model for predicting postoperative overall survival rate in eCCA patients. **(D)** Predictive performance of the nomogram model for 1-year and 3-year prognosis in eCCA patients.

We performed postoperative follow-up for patients with eCCA, and the median survival time was 27 months (**Figure 4A**). Then, we conducted univariate Cox regression analysis on clinical indicators of patients with eCCA to explore their impact on postoperative OS. The results showed that total bilirubin (HR = 1.002, 95% CI: 1.000–1.004, P = 0.024), indirect bilirubin (HR = 1.006, 95% CI: 1.001–1.012, P = 0.016), CA19-9 (HR = 1.001, 95% CI: 1.000–1.002, P = 0.036), cholangiocarcinoma type (HR = 2.314, 95% CI: 1.057–5.066, P = 0.036), and microvascular invasion (HR = 2.441, 95% CI: 1.192–4.998, P = 0.015) were significantly associated with postoperative OS (**Table 2**). In the univariate Cox regression analysis of gut metabolites, we used the logarithmic values of metabolite relative abundances to enhance data interpretation. **Table 3** lists the metabolites that were significantly associated with OS in eCCA patients after surgery. Through multivariate Cox regression analysis, we identified the following factors as independent predictors of OS: preoperative total bilirubin (HR = 1.006, 95% CI: 1.001–1.012, P = 0.017), indirect bilirubin (HR = 0.982, 95% CI: 0.967–0.997, P = 0.018), log (*4-Hydroxyacetophenone* [4-HAP]) (HR = 0.149, 95% CI: 0.037–0.604, P = 0.008), and log (*Palmidrol*) (HR = 8.608, 95% CI: 1.427–51.933, P = 0.019) (**Table 4**). In summary, total bilirubin, indirect bilirubin, and specific metabolites play crucial roles in predicting postoperative OS in patients with eCCA.

**Table 2:** Univariate analysis of clinical indicators affecting the prognosis of patients with eCCA.

Characteristics	HR (95%CI)	*P* value	Characteristics	HR (95%CI)	*P* value
Sex (male)	0.849 (0.399~1.804)	0.669	CEA	1.014 (0.991~1.037)	0.237
Age	1.023 (0.989~1.058)	0.188	Hypertension	1.623 (0.745~3.537)	0.223
BMI	0.957 (0.845~1.083)	0.486	Diabetes	2.018 (0.612~6.651)	0.249
TBIL	1.002 (1.000~1.004)	0.024	cholecystectomized	0.781 (0.186~3.281)	0.736
DBIL	1.002 (1.000~1.005)	0.053	Preoperative biliary drainage	1.042 (0.317~3.429)	0.946
IBIL	1.006 (1.001~1.012)	0.016	HBV-infected	1.112 (0.265~4.664)	0.885
ALP	1.000 (0.999~1.001)	0.763	Anatomical type (dCCA)	2.314 (1.057~5.066)	0.036
GGT	1.000 (0.999~1.000)	0.207	differentiated degree	0.785 (0.356~1.733)	0.549
Total bile acid	1.000 (0.998~1.002)	0.831	TNM_stage	0.750 (0.262~2.148)	0.592
AFP	0.867 (0.622~1.210)	0.403	Microvascular invasion	2.441 (1.192~4.998)	0.015
CA-199	1.001 (1.000~1.002)	0.036	Peripheral nerve invasion	1.038 (0.464~2.321)	0.928

**Table 3:** Univariate analysis of gut metabolites affecting the prognosis of patients with eCCA.

Characteristics	HR (95%CI)	*P* value
log (Oleic Acid)	0.650 (0.433~0.978)	0.039
log (1-Aminocyclopropanecarboxylic Acid)	2.027(1.043~3.941)	0.037
log (Aminomalonic Acid)	0.215 (0.053~0.870)	0.031
log (Prolin)	0.288 (0.098~0.844)	0.023
log (Beta-D-Fructose 6-Phosphate)	1.885 (1.040~3.419)	0.037
log (Aminocaproic Acid)	0.441 (0.226~0.859)	0.016
log (Sorbose)	1.852 (1.057~3.244)	0.031
log (Ala-Gly)	2.415 (1.049~5.556)	0.038
log (D-Xylono-1,5-Lactone)	5.065 (1.048~24.486)	0.044
log (Morphine)	0.553 (0.330~0.926)	0.024
log (4-Hydroxyacetophenone)	0.395 (0.178~0.875)	0.022
log (Mg(18:0/0:0/0:0))	0.054 (0.004~0.712)	0.026
log (Palmidrol)	3.496 (1.296~9.428)	0.013

**Table 4:** Multivariate analysis of factors affecting the prognosis of patients with eCCA.

Characteristics	HR (95%CI)	*P* value
TBIL	1.006 (1.001~1.012)	0.017
IBIL	0.982 (0.967~0.997)	0.018
log (4-Hydroxyacetophenone)	0.149 (0.037~0.604)	0.008
log (Palmidrol)	8.608 (1.427~51.933)	0.019

### 3.6 Predictive value of gut metabolites for the prognosis of patients with resectable eCCA

Based on the results of the Cox regression analysis, we constructed a nomogram model (**Figure 4B**), where higher scores indicate better prognosis. The model achieved a C-index of 0.718, and the calibration curve demonstrated good predictive accuracy (**Figure 4C**). Additionally, the AUCs for predicting 1-year and 3-year prognosis were 0.642 and 0.754, respectively, indicating that the nomogram has moderate efficacy in predicting postoperative survival in patients with eCCA (**Figure 4D**).

## 4 Discussion

This study aimed to explore the characteristics of the gut microbiota and metabolites in patients with eCCA and assess their impact on patient prognosis. We collected fecal samples from eCCA patients and healthy volunteers in Northwest China for 16S rRNA gene sequencing and metabolomics analysis. By comprehensively analyzing the clinical characteristics, gut microbial composition, metabolite profiles, and prognosis of the patients, our study revealed alterations in the gut microbial community and metabolite profiles of eCCA patients. Additionally, we developed a predictive model to evaluate the prognosis of these patients.

Research focusing on the gut microbiota of eCCA patients is relatively limited. In our study, we observed significant differences in beta diversity between the gut microbiota of eCCA patients and healthy controls, with distinct differences at both the phylum and genus levels. These differences suggest that dysbiosis of the gut microbiota may be associated with the occurrence and progression of eCCA. Jan Bednarsch et al. found that 84.2% of patients with perihilar cholangiocarcinoma had biliary bacterial colonization, with common bacteria including *Enterococcus faecalis*, *Enterococcus faecium*, *Enterobacter cloacae*, and *Escherichia coli*, which are also major causes of postoperative abdominal infections (Bednarsch et al., 2021). Furthermore, Di Carlo et al. reported that abnormal proliferation of *Enterobacter* in bile was closely associated with decreased survival rates, suggesting that certain bile bacteria may be risk factors for eCCA progression (Di Carlo et al., 2019). Our LEfSe analysis also showed a significant increase in *Enterobacter* in the gut microbiota of eCCA patients, consistent with the observed enrichment of the same bacteria in bile, indicating that *Enterobacter* may influence eCCA development and prognosis through retrograde biliary infection.

To further explore the relationship between the gut microbiota and the prognosis of patients with eCCA, we analyzed changes in gut metabolites. The results revealed significant alterations in specific gut metabolites, suggesting that these changes may be closely related to the occurrence and progression of eCCA. Through multivariate Cox regression analysis, we found that *4-Hydroxyacetophenone* (4-HAP) and Palmidrol are independent prognostic factors for postoperative overall survival in eCCA patients. Studies have found that 4-HAP can inhibit the adhesion, invasion, and migration of colon cancer cells by activating nonmuscle myosin-2C (NM2C) and altering the organization of actin within the cells, thereby blocking the mechanical program of tumor metastasis. In a mouse model of colon cancer liver metastasis, 4-HAP significantly reduced the metastatic tumor burden (Bryan et al., 2020). In addition, 4-HAP reduces the dissociation of pancreatic cancer cells, induces the formation of cortical actin bundles, and slows down retrograde actin flow, ultimately inhibiting tumor cell metastasis. In a mouse model bearing human pancreatic cancer, 4-HAP significantly reduced the metastatic burden in the liver (Surcel et al., 2019). Therefore, 4-HAP may regulate NM2C and actin organization to suppress tumor cell migration, invasion, and metastasis, making it a potential therapeutic target for cancer treatment. *Palmidrol*, also known as *Palmitoylethanolamide*, is a lipid mediator similar to endocannabinoids with extensive anti-inflammatory, analgesic, antimicrobial, immunomodulatory, and neuroprotective properties. It demonstrates good tolerability and no side effects in both animals and humans (Clayton et al., 2021). Through its ultramicronized form, *Palmidrol* exhibits inhibitory effects on tumors by suppressing colon cancer cell proliferation via the activation of peroxisome proliferator-activated receptor α (PPAR-α) and G protein-coupled receptor 55 (GPR55). Additionally, it induces cell cycle arrest at the G2/M phase by upregulating Cyclin B1/CDK1, leading to DNA fragmentation (Pagano et al., 2021). In conclusion, both 4-HAP and *Palmidrol* exhibit significant anti-tumor effects. This study further highlights their important roles in predicting the prognosis of eCCA, providing new scientific insights into the molecular mechanisms of cancer and potential targets for therapeutic intervention. These findings demonstrate the promising potential of 4-HAP and *Palmidrol* as novel strategies for cancer treatment.

In recent years, studies on microbial metabolites have garnered significant attention, helping to enhance the understanding of disease mechanisms. Previous studies have demonstrated that bile acids can promote the invasive growth of cholangiocarcinoma cells via S1PR2, which is highly expressed in rat and human cholangiocarcinoma cells and tissues (Liu et al., 2014). Additionally, Li et al. identified that metabolic differences between intrahepatic bile duct stones (IBDS) and iCCA are primarily concentrated in the linoleic acid metabolic pathway, with disruptions in this pathway potentially contributing to the malignant transformation of IBDS to iCCA (Li et al., 2022). Chai et al. further revealed the microbial characteristics within iCCA tumors and the antitumor effects of *P. fungorum*, which inhibit tumor growth by regulating alanine, aspartate and glutamate metabolism (Chai et al., 2023). Changes in the gut microbiota not only affect the host’s immune function and inflammatory responses but also alter the production of metabolites, influencing tumor progression and patient prognosis (de Vos et al., 2022). These findings indicate that alterations in the gut microbiota and metabolites reflect metabolic remodeling within the tumor microenvironment, which may have profound implications for tumor development and prognosis.

Multiple factors are associated with the prognosis of eCCA, but studies on the relationship between gut microbiota, metabolites, and prognosis in eCCA remain limited. This study integrated data from multiple levels, including clinical indicators, gut microbiota composition, and metabolite profiles, to uncover their associations and impact on the prognosis of eCCA patients. Previous research has shown that isolated biliary candidiasis may be associated with poor prognosis in patients with unresectable cholangiocarcinoma (Kim et al., 2016). Diagnostic models based on oral and gut microbiota have been developed for the early diagnosis of cholangiocarcinoma (Zhang et al., 2021b; Deng et al., 2022; Rao et al., 2022), although they have not yet been applied to prognosis prediction. In our study, we developed a nomogram model to predict eCCA prognosis, revealing that certain specific gut metabolites were significantly associated with OS. Through Cox regression analysis and model construction, we found that preoperative total bilirubin, indirect bilirubin, and specific metabolites were closely related to postoperative OS, providing new reference indicators for prognosis assessment in eCCA patients. This model offers valuable support for clinical decision-making, helping physicians more accurately assess patient prognosis and develop personalized treatment plans.

However, there are some limitations in this study. Firstly, the sample size is limited, and all data were obtained from a single-center, which may affect the generalizability and applicability of the results. Larger-scale, multi-center studies are needed for further validation. Secondly, given the significant impact of age on the gut microbiota, the notable age differences between the comparison groups may introduce some bias. Age has been shown to affect microbial diversity, composition, and metabolic activity (Sun et al., 2023), which may interfere with the observed differences in gut microbiota and metabolites between the groups. Therefore, the results should be interpreted with caution. To reduce this potential bias, future studies should increase the sample size and perform further stratified subgroup analyses, which may help to draw more reliable conclusions. Additionally, the 16S rRNA sequencing technique used for gut microbiota analysis may miss low-abundance microbes due to insufficient sequencing depth, and the lack of standardization in sample processing and analysis may affect the reproducibility of the results. Furthermore, metabolite detection may be influenced by sample storage conditions and the sensitivity of different detection platforms, potentially leading to the omission of key metabolites or bias in the results. Finally, this study did not fully consider all potential prognostic factors, such as treatment regimens, comorbidities, and lifestyle factors, which may have an impact on the results.

This study comprehensively analyzed the role of gut microbiota and fecal metabolites in cholangiocarcinoma, revealing the potential biological significance of specific microbiota and metabolites in the development of cholangiocarcinoma and patient prognosis. The results further clarified the abnormal characteristics of gut microbiota composition in cholangiocarcinoma patients, identifying key microbiota and metabolites that may affect bile acid metabolism and the tumor microenvironment, and validating their predictive value for postoperative survival. These findings provide important insights for early screening, postoperative monitoring, and personalized treatment of cholangiocarcinoma, and offer scientific evidence for exploring new therapeutic targets and intervention strategies.

## Data Availability

The datasets presented in this study can be found in online repositories. The names of the repository/repositories and accession number(s) can be found in the article/**Supplementary Material**. The 16S rRNA data used in this study are available in the NCBI database under BioProject accession code PRJNA1183424.
