# Supplementary figures and images for: Characteristics of gut microbiota and metabolites in extrahepatic cholangiocarcinoma and their prognostic value for resectable lesions
Source: Front Cell Infect Microbiol. 2025 Feb 14;15:1523863. doi: 10.3389/fcimb.2025.1523863 (PMC11868125; doi:10.3389/fcimb.2025.1523863)

$R^2=(0.0,0.771)$   $Q^2=(0.0,-0.624)$

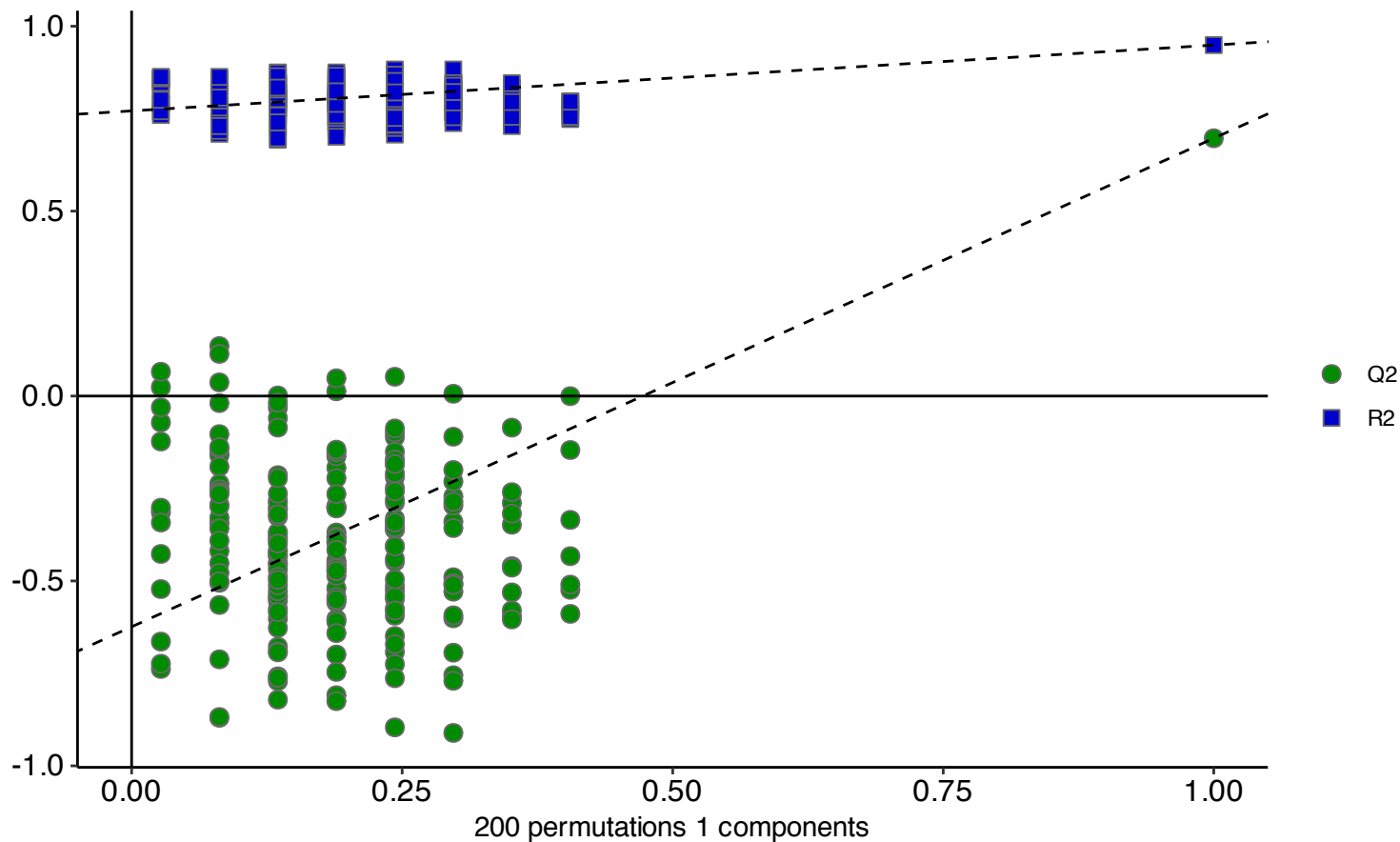

Supplement: Supplementary file 1 [file DataSheet1.pdf]
